# D-Mannose prevents bone loss under weightlessness

**DOI:** 10.1186/s12967-022-03870-1

**Published:** 2023-01-09

**Authors:** Ranli Gu, Hao Liu, Menglong Hu, Yuan Zhu, Xuenan Liu, Feilong Wang, Likun Wu, Danyang Song, Yunsong Liu

**Affiliations:** 1 Department of Prosthodontics, National Clinical Research Center for Oral Diseases, National Engineering Laboratory for Digital and Material Technology of Stomatology, Beijing Key Laboratory of Digital Stomatology, Peking University School and Hospital of Stomatology, Beijing, 100081 China; 2 The Central Laboratory, National Clinical Research Center for Oral Diseases, National Engineering Laboratory for Digital and Material Technology of Stomatology, Beijing Key Laboratory of Digital Stomatology, Peking University School and Hospital of Stomatology, Beijing, 100081 China

**Keywords:** D-mannose, Bone, UTI, Osteoclast, Cell fusion, Weightlessness

## Abstract

**Background:**

Astronauts undergo significant microgravity-induced bone loss during space missions, which has become one of the three major medical problems hindering human's long-term space flight. A risk-free and antiresorptive drug is urgently needed to prevent bone loss during space missions. D-mannose is a natural C-2 epimer of D-glucose and is abundant in cranberries. This study aimed to investigate the protective effects and potential mechanisms of D-mannose against bone loss under weightlessness.

**Methods:**

The hind legs of tail-suspended (TS) rats were used to mimic weightlessness on Earth. Rats were administered D-mannose intragastrically. The osteoclastogenic and osteogenic capacity of D-mannose in vitro and in vivo was analyzed by micro-computed tomography, biomechanical assessment, bone histology, serum markers of bone metabolism, cell proliferation assay, quantitative polymerase chain reaction, and western blotting. RNA-seq transcriptomic analysis was performed to detect the underlying mechanisms of D-mannose in bone protection.

**Results:**

The TS rats showed lower bone mineral density (BMD) and poorer bone morphological indices. D-mannose could improve BMD in TS rats. D-mannose inhibited osteoclast proliferation and fusion in vitro, without apparent effects on osteoblasts. RNA-seq transcriptomic analysis showed that D-mannose administration significantly inhibited the cell fusion molecule dendritic cell-specific transmembrane protein (DC-STAMP) and two indispensable transcription factors for osteoclast fusion (c-Fos and nuclear factor of activated T cells 1 [NFATc1]). Finally, TS rats tended to experience dysuria-related urinary tract infections (UTIs), which were suppressed by treatment with D-mannose.

**Conclusion:**

D-mannose protected against bone loss and UTIs in rats under weightlessness. The bone protective effects of D-mannose were mediated by inhibiting osteoclast cell fusion. Our findings provide a potential strategy to protect against bone loss and UTIs during space missions.

**Supplementary Information:**

The online version contains supplementary material available at 10.1186/s12967-022-03870-1.

## Background

Serious weightlessness-induced bone loss occurs in astronauts during space flight, which leads to a significant increase in the risk of fractures and nephrolith and causes irreversible damage to the skeletal system [1–3], making it one of the three major medical problems hindering human long-term space travel [4]. Bone mineral density (BMD) decreases by approximately 1–2% after 1 month in space, equivalent to the annual bone loss in postmenopausal women [5]. Even when astronauts return to Earth, reversal of bone loss is difficult [6]. This rapid and substantial bone loss is a major challenge that must be addressed to ensure that astronauts remain healthy during space missions. Therefore, the search for safe and effective protective measures against weightlessness-induced bone loss has been a constant focus of space medical research.

Normally, osteoblasts form new bone while osteoclasts absorb bone, thus bone mass usually remains balanced. Mechanical stimulation is crucial for the development and maintenance of bone structure. Mechanical loading expedites bone formation, conversely, the absence of mechanical stimulation reduces bone mass [7]. Numerous studies have demonstrated that the lack of mechanical stimulation is the dominant cause of bone loss in space [8].

Space travel also leads to reduced immune competence and a potential increase in the risk of infections [9–11]. Because space flight results in altered nutrition intake by crew members and further affects immune responses [12], nutritional countermeasures are needed to enhance immune responses. Numerous studies have demonstrated that microbes become more virulent and more resistant to antibiotics during exposure to microgravity [13, 14], thereby hindering the treatment of infections in space.

Multiple interventions have been explored to prevent bone loss in space, including physical exercise and related medical interventions. However, the tested compounds had side effects such as digestive and renal disturbance, and hepatic dysfunction [15–17]. There are strict limits regarding substances that can be used by astronauts during space missions, and drug interventions are not routinely used in space. Therefore, the addition of functional foods or healthcare products to a diet may be helpful.

An extensive literature search indicated that various natural fruit juices including cranberry [18, 19], blueberry [20], coconut [21], and citrus [22] have protective effects on bone. Among these, cranberries have various beneficial effects such as preventing certain types of cancers [23], cardiovascular diseases [24], neurological disorders [25], and infectious diseases especially urinary tract infections (UTIs) [26, 27]. The main active components in cranberries are quercetin, myricetin, kaempferol [28], anthocyanins [29], and D-mannose.

D-mannose, a popular nutritional and health food supplement, is a natural C-2 epimer of D-glucose and is abundant in cranberries [30]. A safe supraphysiological dose of D-mannose has beneficial effects on individual health. D-mannose is widely used as a treatment for a congenital disorder of glycosylation [31, 32] and recurrent UTIs [33, 34]. Numerous studies have shown that D-mannose binds to the cilia of *Escherichia coli*, preventing bacterial attachment to the urothelium in UTIs [35–38]. D-mannose does not cause bacterial resistance because it is a non-antibiotic compound and an anti-inflammatory substance; these properties may be useful for the treatment of UTIs in astronauts during space missions. Furthermore, D-mannose suppresses T cell-mediated immunopathology [39] and macrophage IL-1β production [40]. Recently, we reported that D-mannose could attenuate senility-induced and estrogen-deficient bone loss in mice [41]. Moreover, we revealed that D-mannose mediates bone protection via regulatory T-cell proliferation and anti-inflammatory effects [41]. Furthermore, Yang et al. reported that D-mannose attenuated alveolar bone loss in a periodontitis mouse model by regulating the anti-inflammatory effects of amino acids [42]. However, the specific mechanism underlying D-mannose-mediated bone protection requires further investigation.

The tail-suspended (TS) rat model established by Jee et al. is a widely accepted animal model for mimicking weightlessness [43]. Rats placed in a head-down tilt position exhibit bone mass reduction and a decrease in bone mechanical strength [44, 45]. Therefore, we used the TS rat model to simulate weightlessness on Earth and aimed to investigate the protective effects and potential mechanisms of D-mannose against bone loss under weightlessness.

## Methods

### Animals

The Animal Care and Use Committee of Peking University Health Science Center approved all animal experiments (approval number: LA2021006; Beijing, China). The rats were randomly divided into five groups: Sham: rats with normal body position and normal diet; TS: tail-suspended rats administered a daily dose of 2 mL of sterile water by gavage; Cranberry: tail-suspended rats administered a daily dose of 2 mL of cranberry juice by gavage; Blueberry: tail-suspended rats administered a daily dose of 2 mL of blueberry juice by gavage; and Raspberry: tail-suspended rats administered a daily dose of 2 mL of raspberry juice by gavage (Additional file 1: Fig. S1). The rats were also randomly divided into four groups: Sham: as described above; Sham + Man: rats with normal body position administered a daily dose of 1.1 M D-mannose by gavage; TS: tail-suspended rats administered a daily dose of sterile water by gavage; and TS + Man: tail-suspended rats administered a daily dose of 1.1 M D-mannose by gavage. The rats were suspended by tail to maintain a head-down tilt of 30° with the hind legs elevated for 28 days. The rats were allowed a 360° range of movement to facilitate free movement in the cage (Additional file 1: Fig. S1 a, b).

### Preparation of three fruit juices

Fresh cranberries, blueberries, and raspberries were harvested in the Greater Khingan region and frozen after homogenization. Each fruit mixture was centrifuged at 20,000 g for 10 min and filtered to remove insoluble particles. Aliquots of the three types of juice were stored at − 20 °C until use. Juice was intragastrically administered to rats at a dose of 500 mg/kg body weight per day for 28 days.

### Serum bone metabolic markers

After the collection of blood from the vena cava, blood samples were centrifuged at 3000 rpm for 15 min to isolate serum. Serum levels of procollagen type 1 N-terminal propeptide (P1NP) and C-terminal telopeptide of type I collagen (CTX-I) were measured using an enzyme immunoassay kit (Rat/Mouse P1NP Enzyme Immunoassay Kit, Immunodiagnostic Systems Ltd., Boldons, UK) and a solid phase immunofixed enzyme activity assay (RatTRAP test, Immunodiagnostic Systems Ltd.).

### Micro-computed tomography (CT) analysis

To investigate differences in bone mass and microarchitecture among the groups, the micro-computed tomography Inveon MM system (Siemens, Munich, Germany) was used to analyze bone specimens. Briefly, images of rat proximal femur, centrum, and mandible were scanned with 8.82 μm pixel size, 220 μA current, 60 kV voltage, and 1500 ms exposure time [46]. Bone histomorphometry parameters and BMD (1–2 mm distal to the proximal epiphysis) were calculated using an Inveon Research Workplace (Siemens) in accordance with common guidelines [47].

### Hematoxylin and eosin (H&E) and tartrate-resistant acid phosphatase (TRAP) staining of femurs

After 1 week of fixation in 4% paraformaldehyde, femur specimens were immersed in 10% ethylenediaminetetraacetic acid decalcification solution and placed on a 37 °C shaker for 2 weeks. The decalcification solution was changed every 2 days. Thick Sections. (5 mm) were stained with H&E to observe morphology. TRAP staining was conducted to detect osteoclasts (defined as red-wine colored cells with multiple nuclei) [48] using a TRAP kit (Sigma-Aldrich, St. Louis, MO, USA).

### Cell culture

RAW264.7 cells were purchased from the American Type Culture Collection (Manassas, VA, USA) and divided into four groups: OCM (osteoclast induction medium, 100 ng/mL RANKL in α-Minimum Essential Medium [Gibco BRL/Invitrogen, Carlsbad, CA, USA] containing 10% fetal bovine serum [Biowest, France]); OCM + Man (100 ng/mL RANKL plus 25 mM D-mannose); OCM + Glu (100 ng/mL RANKL plus 25 mM glucose); and OCM + Fru (100 ng/mL RANKL plus 25 mM fructose). Rat bone marrow monocytes (rBMMs) were extracted from femurs and flushed with a syringe. rBMMs were collected by centrifugation and incubated with red blood cell lysis buffer for 10 s at room temperature, as previously described [49]. rBMMs were cultured in vitro without RANKL for only 4 days to represent the in vivo cellular state.

### Cell proliferation

RAW264.7 cells and rBMMs were seeded at 10,000 cells/well in 24-well plates. Cell Counting Kit-8 (Dojindo Laboratories, Kumamoto, Japan) was used in accordance with the manufacturer’s instructions, and the light absorbance (optical density) at 450 nm of the formazan dye product was used to measure cell proliferation.

### TRAP staining and cell activity assay

Osteoclastic differentiation of cells was evaluated using TRAP staining with the Leukocyte Acid Phosphatase Kit (Sigma-Aldrich) and TRAP activity assay (TRAP Assay Kit; Takara, Shiga, Japan). Cultured RAW264.7 cells and rBMMs were fixed in 3.7% paraformaldehyde for 10 min, treated with 0.1% Triton X-100 in phosphate-buffered saline at room temperature for 5 min, and rinsed 3 times with deionized water. Finally, cells were incubated with 0.01% naphthol AS-MX phosphate and 0.05% fast red violet LB salt in 50 mM sodium tartrate and 90 mM sodium acetate (PH = 5.0) for 1 h at 37 °C, then rinsed 3 times with deionized water. TRAP activity was measured using the cell culture supernatant after staining.

### Analysis of in vitro bone resorption

RAW264.7 cells and rBMMs were seeded on sterilized bovine bone slices (Immunodiagnostic Systems Inc., Boldon, UK) placed in 48-well plates. After 4 days, each well was washed with deionized water. To measure the depth and area of resorption pits, each disc was observed and analyzed using a scanning electron microscope (S-4800; Hitachi, Tokyo, Japan).

### Staining and quantification of alkaline phosphatase (ALP) and Alizarin red S (ARS) in rMSCs

To examine the osteogenic effect of D-mannose, rat bone marrow stroma cells (rMSCs) were extracted as previously described [50]. For osteo-induction assays, 10 mM β-glycerophosphate, 10 nM dexamethasone, and 50 μg/mL ascorbic acid (Sigma-Aldrich) were added to low-glucose Dulbecco’s modified Eagle medium containing 10% fetal bovine serum, 100 U/mL penicillin, and 100 mg/mL streptomycin (Gibco BRL). The cells were subjected to osteo-induction for 14 days, then stained with a nitroblue tetrazolium/5-bromo-4-chloro-3-indolyl phosphate staining kit (CoWin Biotech, China) or 2% ARS staining solution (Sigma-Aldrich). Absorbance was measured at 520 nm and the ALP activity was calculated. For ARS quantification, cells were completely dissolved and mineral accumulation was measured by absorbance at 562 nm.

### RNA-seq transcriptomic analysis

RNA quantity and integrity were assessed using the RNA Nano 6000 Assay Kit of the Bioanalyzer 2100 system (Agilent Technologies, Santa Clara, CA, USA). Normalization and quantification of miRNA were conducted by comparison to miRBase. The edgeR program was used to identify differentially expressed genes; genes with an expression fold change > 1.5 were defined as differentially expressed. Gene Ontology (GO) enrichment analysis of differentially expressed genes was conducted by Integrative Genomics Viewer (IGV) (Wayen Biotechnologies, Shanghai, China).

### Quantitative polymerase chain reaction (qPCR)

Total RNA was extracted using TRIzol reagent (Invitrogen), then used to synthesize first-strand cDNA with the Prime Script RT Reagent Kit (Takara, Tokyo, Japan). Next, qPCR was performed using the 7500 Real-Time PCR Detection System (Applied Biosystems, Foster City, CA, USA). The following thermocycler protocol was used: 95 °C for 10 min, followed by 40 cycles of 95 °C for 15 s and 60 °C for 1 min. The primers used for qPCR are shown in Table 1.

**Table 1:** Sequences of the primers used in qRT-PCR

	Forward primer (5ʹ to 3ʹ)	Reverse primer (5ʹ-3ʹ)
mGap	TGCACCACCAACTGCTTAGC	GGCATGGACTGTGGTCATGAG
mRank	GGCTTACCTGCCCAGTCTCATC	AAGCATCATTGACCCAATTCCAC
mRankl	GCAGCATCGCTCTGTTCCTGTA	CCTGCAGGAGTCAGGTAGTGTGTC
mMmp9	GCCCTGGAACTCACACGACA	TTGGAAACTCACACGCCAGAAG
mCtk	TGTATAACGCCACGGCAAA	GGTTCACATTATCACGGTCACA
mTrap	CAGCAGCCAAGGAGGACTAC	ACATAGCCCACACCGTTCTC
mNfatc1	TGCTCCTCCTCCTGCTGCTC	CGTCTTCCACCTCCACGTCG
mC-fos	ATGGGCTCTCCTGTCAACAC	GGCTGCCAAAATAAACTCCA
mDc-stamp	CTAGCTGGCTGGACTTCATCC	TCATGCTGTCTAGGAGACCTC
rGap	CGGACAGGATTGACAGATTGATAGC	TGCCAGAGTCTCGTTCGTTATCG
rRank	TCGGGTTCCATAAAGTCAG	CTGAAGCAAATGTTGGCGTA
rRankl	TCGGGTTCCCATAAAGTCAG	CTGAAGCAAATGGCGGTA
rMmp9	GCGAGACACTAAAGGCCAT	ATGGCCTTTAGTGTCTCGC
rCtk	GGCTCTGCCGTTGTTTCTCT	AAGGTGCTTTGGGAATCTGC
rTrap	GCAGAGACTCTTTCGGGCTTT	CATTCATGGTGCAGCTTATCGA
rAlp	GGCTCTGCCGTTGTTTCTCT	AAGGTGCTTTGGGAATCTGC
rRunx2	ATGAGGACCCTCTCTCTGCTC	CTAAACGGTGGTGCCATAGAT
rOcn	AGCGGACGAGGCAAGAGTTT	CTGTCTGTGCCTTCTTGGTTCC
rSp7	GGCTCTGCCGTTGTTTCTCT	AAGGTGCTTTGGGAATCTGC
rNfatc1	CTCTCAGGACAATGCAGTGCTGA	ATCCAGGTCACACATTCCAGCA
rC-fos	ACTTCCCCAGCCCTTACTACCG	TCAGCACATAGCCCACACCG
rDc-stamp	TCTCAGTGTGTCTGAGACTTG	GACTGTGTTGCTCATAGATCATC

### Immunofluorescence staining

RAW264.7 cells and rBMMs plated on sterile glass coverslips were fixed for 10 min with 3.7% formaldehyde, then permeabilized in 0.1% Triton X-100. Next, cells were incubated for 1 h at room temperature with anti-fluorescein isothiocyanate antibody (1:1000; Abcam, Cambridge, UK). After cells had been washed with Tris-buffered saline, they were incubated for 1 h at room temperature with 4′,6-diamidino-2-phenylindole and mounted on glass slides. Images were acquired using a fluorescence microscope (Olympus, Tokyo, Japan).

### Western blotting

RAW264.7 cells and rBMMs were lysed in radioimmunoprecipitation assay buffer (Sigma-Aldrich) and centrifuged at 4 °C for 30 min at 14,000 rpm. The supernatant was collected, and the extracted proteins were subjected to 10% sodium dodecyl sulfate–polyacrylamide gel electrophoresis (CWBIO, Beijing, China), then transferred to polyvinylidene fluoride membranes (Millipore, Billerica, MA, USA). The membranes were incubated overnight with anti-c-Fos, anti-NFATc1, anti-DC-STAMP, or anti-glyceraldehyde-3-phosphate dehydrogenase antibodies (1:1,000; Abcam). Then, the membranes were incubated with secondary antibody solution at room temperature. Immunoreactive protein bands were detected using an ECL kit (CWBIO, Beijing, China).

### Statistical analysis

The Shapiro Wilk was used to test the normal distribution of the data in IBM SPSS Statistics v20.0 software (SPSS Inc, Chicago, IL, USA), and the P-values greater than 0.05 were considered that the data are normally distributed. One-way analysis of variance (ANOVA) was used for statistical analyses and the P-values less than 0.05 were considered to indicate statistical significance. Data are indicated as mean ± standard deviation (SD).

## Results

### Cranberries protected against in vivo bone loss in TS rats

**Fig. 1:**
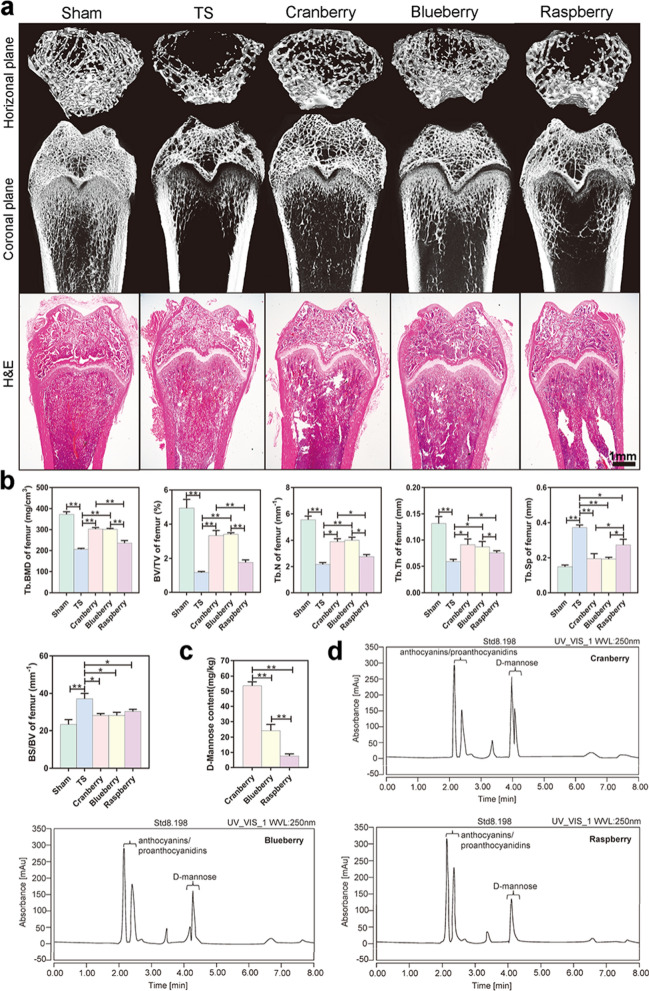
Cranberries protected against in vivo bone loss in rats exposed to simulated microgravity. **a** Representative images of femurs in horizontal plane and coronal plane reconstructed by micro-CT or slicing of H&E staining of rat femurs, bar represents 1 mm. **b** BMD and bone microarchitecture were measured in the distal femur using micro-CT. **c** The content of D-mannose measured by HPLC in cranberry, blueberry and raspberry. Data are expressed as mean ± SD. **d** Chromatogram of HPLC of D-mannose in cranberry, blueberry and raspberry

To investigate whether cranberries could attenuate bone loss under weightlessness, we designed a rat suspension device (Additional file 1: Fig. S1a) and suspended the rats for 28 days (Additional file 1: Fig. S1b), during which the rats were administered pure cranberry, blueberry, or raspberry fruit juice (Additional file 1: Fig. S1c) by gavage. Then, the rats in all five groups (Sham, TS, Cranberry, Blueberry, and Raspberry) were sacrificed and their bones were analyzed by micro-CT and histological staining (Fig. 1a).

TS rats exhibited lower BMD in trabecular bone (Tb.BMD), and lower bone volume/total volume ratio (BV/TV), trabecular numbers (Tb.N), and trabecular thickness (Tb.Th) in femurs compared with rats in the Sham group (*P* < 0.001) (Fig. 1b). By contrast, the bone surface area/bone volume ratio (BS/BV) as well as trabecular separation (Tb.Sp) of femurs were significantly greater in the TS group than in the Sham group (*P* < 0.001, respectively). These results indicated significant bone loss in TS rats.

Tb.BMD and BV/TV in femurs in the Cranberry and Blueberry groups as well as Tb.N in Blueberry group were significantly greater than in the TS group (*P* < 0.001, respectively). Tb.N in Cranberry group was much higher than in the TS group (*P* = 0.023). However, the Raspberry group showed no changes indicative of an osteoprotective effect, compared to the TS group. Furthermore, Tb.BMD and BV/TV in femurs were greater in the Cranberry and Blueberry groups than in the Raspberry group (*P* < 0.001, respectively). Tb.N and Tb.Th in femurs were also higher in the Cranberry and Blueberry groups than in the Raspberry group (*P* = 0.037, Cranberry *vs* Raspberry; *P* = 0.041, Blueberry *vs* Raspberry). By contrast, Tb.Sp in femurs was significantly reduced in the Cranberry and Blueberry groups, compared to the TS group (*P* < 0.001). BS/BV in femurs was obviously reduced in the Cranberry and Blueberry groups, compared to the TS group (*P* = 0.019, Cranberry *vs* Raspberry; *P* = 0.022, Blueberry *vs* Raspberry). No obvious differences in osteo-protection were observed between the Cranberry and Blueberry groups (Fig. 1b).

High-performance liquid chromatography (HPLC) was performed to identify the osteoprotective components in cranberries. The D-mannose content was highest in cranberries, followed by blueberries and raspberries (*P* < 0.001) (Fig. 1c). HPLC chromatograms of D-mannose in the three fruits are shown in Fig. 1d. Interestingly, while the contents of anthocyanins and proanthocyanidins were relatively high in the three fruits (Additional file 1: Fig. S2), there were no significant differences in their contents.

### D-mannose protected against in vivo bone loss and UTIs in TS rats

Bones from four groups of rats (Sham, Sham + Man [Sham + D-mannose administration for 28 days], TS, and TS + Man [TS + D-mannose administration for 28 days]) were collected for micro-CT analysis and histological staining. During the 4-week suspension period prior to sacrifice, the rat weights were lower in the TS group than in the Sham group; however, the rat weights did not differ between the TS and TS + Man groups (Additional file 1: Fig. S1e).

**Fig. 2:**
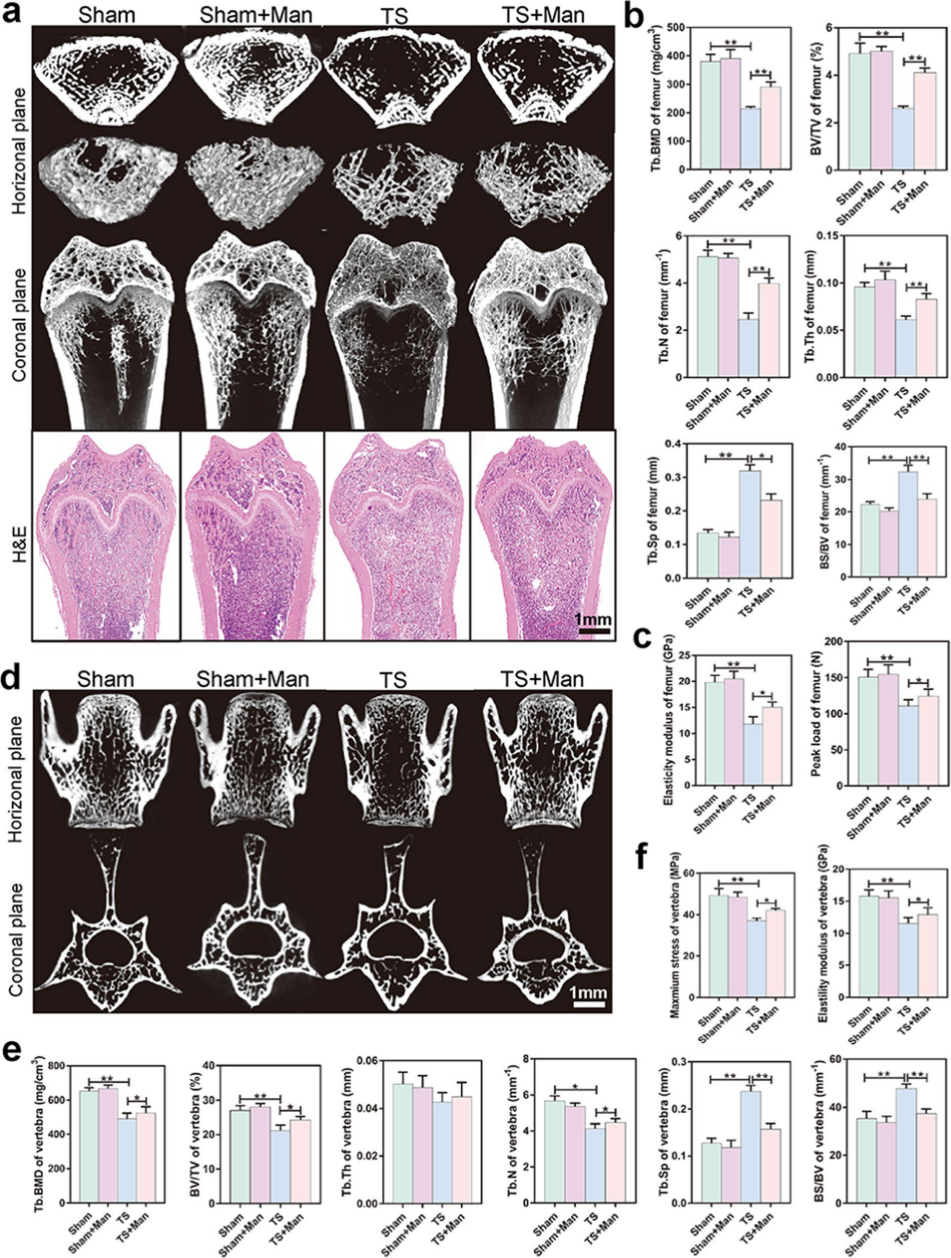
D-mannose protected against in vivo bone loss in rats exposed to simulated microgravity. **a** Representative micro-CT images of femurs in horizontal plane and coronal plane or slicing of H&E staining of femurs, bar represents 1 mm. **b** BMD and bone microarchitecture of femurs were measured in the distal femur using micro-CT. **c** The biomechanical parameters of peak load and elasticity modulus of femurs were evaluated by three-point flexural test. **d** Representative images of vertebrae in horizontal plane and coronal plane reconstructed by micro-CT, bar represents 1 mm. BMD and bone microarchitecture of vertebrae were measured using micro-CT. **e** BMD and bone microarchitecture of vertebrae were measured in the distal femur using micro-CT. **f** The biomechanical parameters of maximum stress and elasticity modulus of vertebrae were evaluated by three-point flexural test. Data are expressed as mean ± SD

Consistent with the findings in the juice gavage experiment, the Tb.BMD, BV/TV, Tb.N, and Tb.Th in femurs were lower in the TS group than in the Sham group (*P* < 0.001) (Fig. 2a, b), similar to the findings in the Tb.BMD and BV/TV for vertebrae (*P* < 0.001) as well as Tb. N for vertebrae (*P* = 0.026) (Fig. 2d, e). By contrast, the BS/BV in femurs and vertebrae, and Tb.Sp in vertebrae were significantly greater in the TS group than in the Sham group (*P* < 0.001). Tb.Sp in femurs was significantly higher in the TS group than in the Sham group (*P* = 0.034). Notably, the mandibular BMD was similar in the TS and Sham groups (Additional file 1: Fig. S3).

D-mannose administration significantly increased Tb.BMD, BV/TV, Tb.Th, and Tb.N in femurs in the TS + Man group, compared with the TS group (*P* < 0.001). With the exception of Tb.Th, D-mannose administration also significantly increased Tb.BMD, BV/TV and Tb.N in vertebrae in the TS + Man group compared with the TS group (*P* = 0.045, *P* = 0.028, *P* = 0.036, respectively). By contrast, D-mannose administration significantly reduced BS/BV in femurs and vertebrae as well as Tb.Sp in vertebrae in the TS + Man group, compared to the TS group (*P* < 0.001). Furthermore, D-mannose administration significantly reduced Tb.Sp in femurs in the TS + Man group, compared with the TS group (*P* = 0.033). Consistent with the histomorphometry findings, the peak load and elasticity modulus of femurs and vertebrae were significantly lower in the TS group than in the Sham group (*P* < 0.001) (Fig. 2c, f). Furthermore, D-mannose administration significantly increased the peak load and elasticity modulus of femurs (*P* = 0.018, *P* = 0.032, respectively) and vertebrae (*P* = 0.041, *P* = 0.039, respectively) in the TS + Man group, compared with the TS group (Fig. 2c, f). H&E staining of femur sections showed similar results (Fig. 2a). The above findings indicated that D-mannose could prevent disuse-induced bone loss in TS rats exposed to simulated microgravity in vivo.

H&E staining revealed a large zone of infiltrating inflammatory cells in renal tissue from rats in the TS group. H&E staining of the urethral tissue from rats in the TS group also showed scattered hemorrhagic foci (Additional file 1: Fig. S4a). Additionally, the numbers of white blood cells and blood neutrophils in urine were significantly greater in the TS group than in the Sham group (*P* < 0.001) (Additional file 1: Fig. S4b), indicating that rats in the TS group developed UTIs because of dysuria induced by the sustained head-down tilt position. Furthermore, we did not observe inflammatory infiltration in sections of liver, spleen, or kidney from rats in the Sham + Man or TS + Man groups (Additional file 1: Fig. S5). These results indicate that D-mannose is a promising and safe alternative to antibiotics for the treatment of UTIs in space.

### D-mannose suppressed osteoclastogenesis but did not promote osteogenesis in vivo

**Fig. 3:**
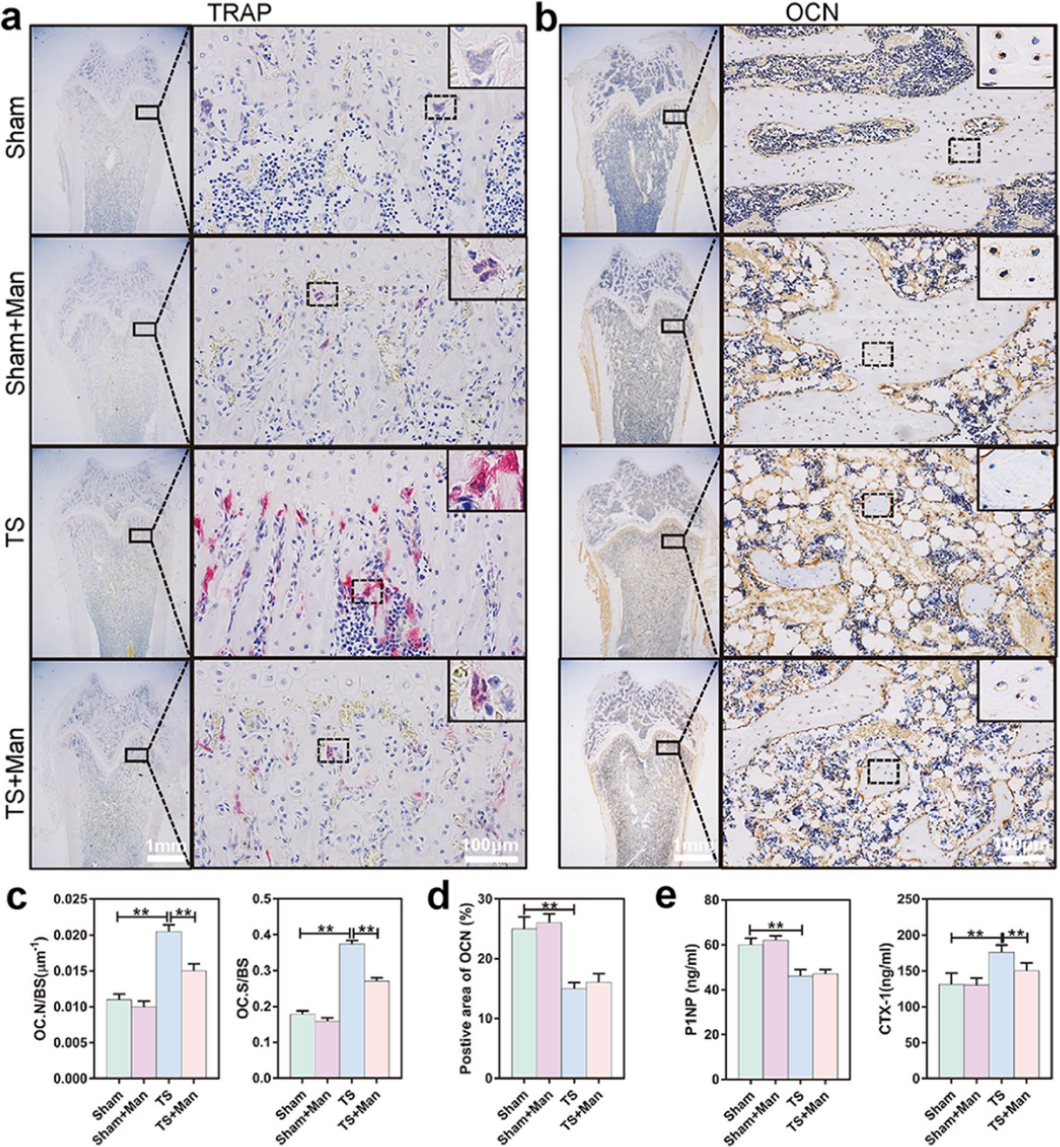
D-mannose suppressed osteoclastogenesis but did not promote osteogenesis in vivo. **a** Representative TRAP staining images of femurs, bar represents 1 mm and 100 μm, respectively. The cells with more than three nuclei and stained as claret-red color are osteoclasts. **b** Immunohistochemical staining of OCN were used to observe osteogenesis in distal femur. The black dotted rectangles indicate positive areas while solid rectangles refer to a higher magnification of immunoreactivity positive areas, bar represents 1 mm and 100 μm, respectively. **c** The number of osteoclasts per bone unit area (OC. N/BS) and the percent of surfaces area of osteoclasts (OC. S/BS) were counted. **d** Positive areas of OCN were counted. **e** The content of CTX1 and P1NP in rat serum. Data are expressed as mean ± SD

Femurs from TS rats exhibited greater numbers of osteoclasts (i.e., TRAP-positive cells with > 3 nuclei) [48] in TRAP staining results (Fig. 3a). Imaging revealed that the number of osteoclasts was smaller in the TS + Man group than in the TS group. Furthermore, the ratios of osteoclast numbers to bone surface area and osteoclast surface area to bone surface area were significantly lower in the TS + Man group than in the TS group (*P* < 0.001) (Fig. 3c). These results indicated that bone loss in rats exposed to simulated microgravity was caused by osteoclast activity in vivo, and D-mannose inhibited this osteoclast activity under disuse conditions.

Immunohistochemical staining of osteocalcin (OCN) was also conducted to observe osteogenesis in femurs. Femurs from rats in the TS group exhibited less OCN expression, compared to femurs from rats in the Sham group. However, D-mannose did not significantly influence OCN expression (Fig. 3b). Quantitative immunohistochemistry analyses of OCN expression in femurs showed similar results in all four groups (Fig. 3d).

The level of C-terminal telopeptide of collagen type 1 (CTX-1), a serum marker of osteoclast absorption [51], was obviously lower in the TS + Man group than in the TS group (*P* < 0.001). However, there was no distinct difference in the serum level of procollagen type I N-terminal propeptide (P1NP), an osteogenic marker (Fig. 3e). Analysis of these serum markers further confirmed that D mannose inhibited osteoclastogenesis, but had no significant effect on osteogenesis in vivo.

### D-mannose suppressed osteoclast proliferation and bone resorption capacity in vitro

The above results showed that D-mannose inhibited osteoclast formation but did not significantly influence osteogenesis in vivo.

**Fig. 4:**
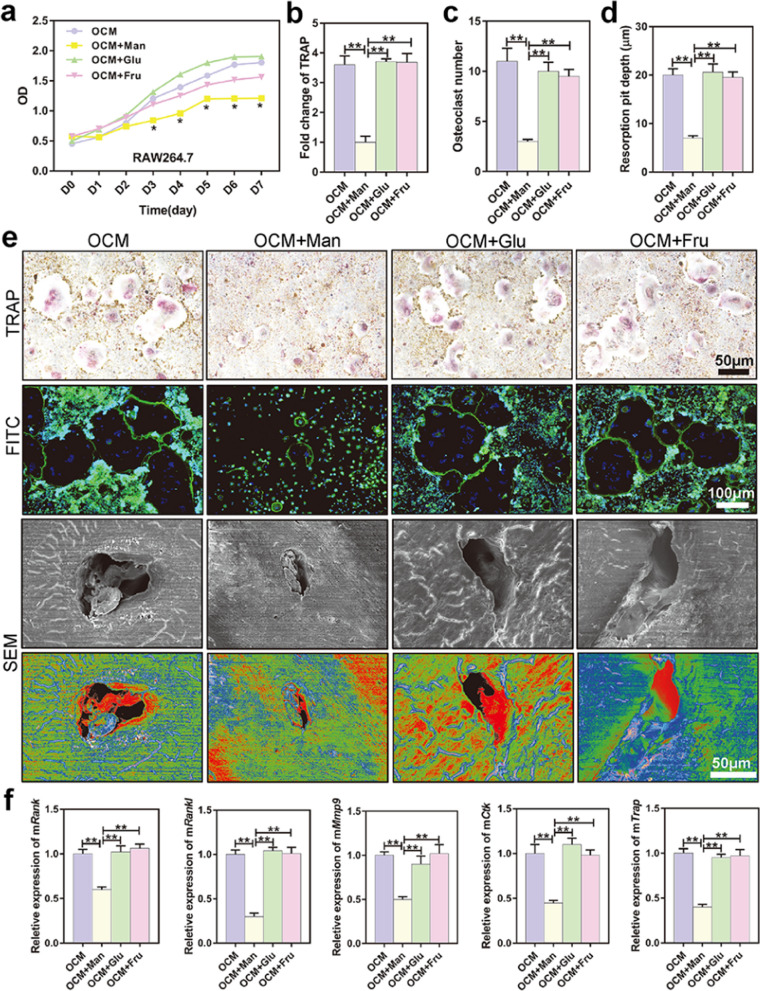
D-mannose suppressed osteoclasts proliferation and bone resorption capacity in vitro. **a** RAW264.7 cells were cultured and induced to osteoclast-like cells (OCLs) by Rankl. The CCK-8 proliferation curve of OCLs. **b** Quantification of TRAP staining results. **c** The number of osteoclasts were counted in FITC immunofluorescence staining. **d** Bone pit absorption depth of fresh sterile bovine bone grinding in SEM. **e** Representative TRAP staining images of OCLs. Pictures on the second line are representative FITC immunofluorescence staining of OCLs. The blue ones are the nuclei stained by DAPI and the green ones are the cell membranes stained by FITC. The giant cells with more than three nuclei are osteoclasts. Pictures on the third and fourth line are representative SEM images of bone grinding slice on which OCLs grow. Bar represents 100 μm and 50 μm, respectively. **f** Relative expression of osteoclastogenesis-relating genes including *mRank*, *mRankl*, *mMmp9*, *mCtk*, and *mTrap*. Data are expressed as mean ± SD

RAW264.7 cells were used to study osteoclast inhibition by D-mannose. The cells were divided into four groups according to the culture medium conditions used to induce differentiation into osteoclast-like cells: OCM (culture medium only, including 100 ng/mL RANKL), OCM + Man (culture medium + 25 mM D-mannose), OCM + Glu (culture medium + 25 mM glucose), and OCM + Fru (culture medium + 25 mM fructose). As shown in Fig. 4a, the proliferation of osteoclast-like cells was inhibited in the OCM + Man group, compared to the OCM, OCM + Glu, and OCM + Fru groups (*P* = 0.024, *P* = 0.019, *P* = 0.013, *P* = 0.015, *P* = 0.037, respectively). TRAP-positive claret-red multinucleated osteoclast-like cells with > 3 nuclei were regarded as osteoclasts [52]. As shown in Fig. 4b–e and Additional file 1: Fig. S6, TRAP and fluorescein isothiocyanate (FITC) staining, as well as scanning electron microscopy (SEM) revealed that the number of TRAP-stained osteoclasts was significantly decreased and resorption pit depth was shallower in the OCM + Man group than in the other three groups (*P* < 0.001). Furthermore, the levels of osteoclastogenesis-associated mRNAs (e.g., *Rank*, *Rankl*, *Mmp9*, *Ctk*, and *Trap*) were lower in the OCM + Man group than in the other three groups (*P* < 0.001) (Fig. 4f). However, the presence of glucose or fructose at the same concentration did not inhibit osteoclast formation or absorption.

### D-mannose suppressed osteoclast proliferation and bone resorption capacity in vivo

To further confirm the osteoclast inhibitory effects of D-mannose, rBMMs from each of the four groups were extracted and cultured in vitro for 4 days to represent the in vivo cellular state*.*

**Fig. 5:**
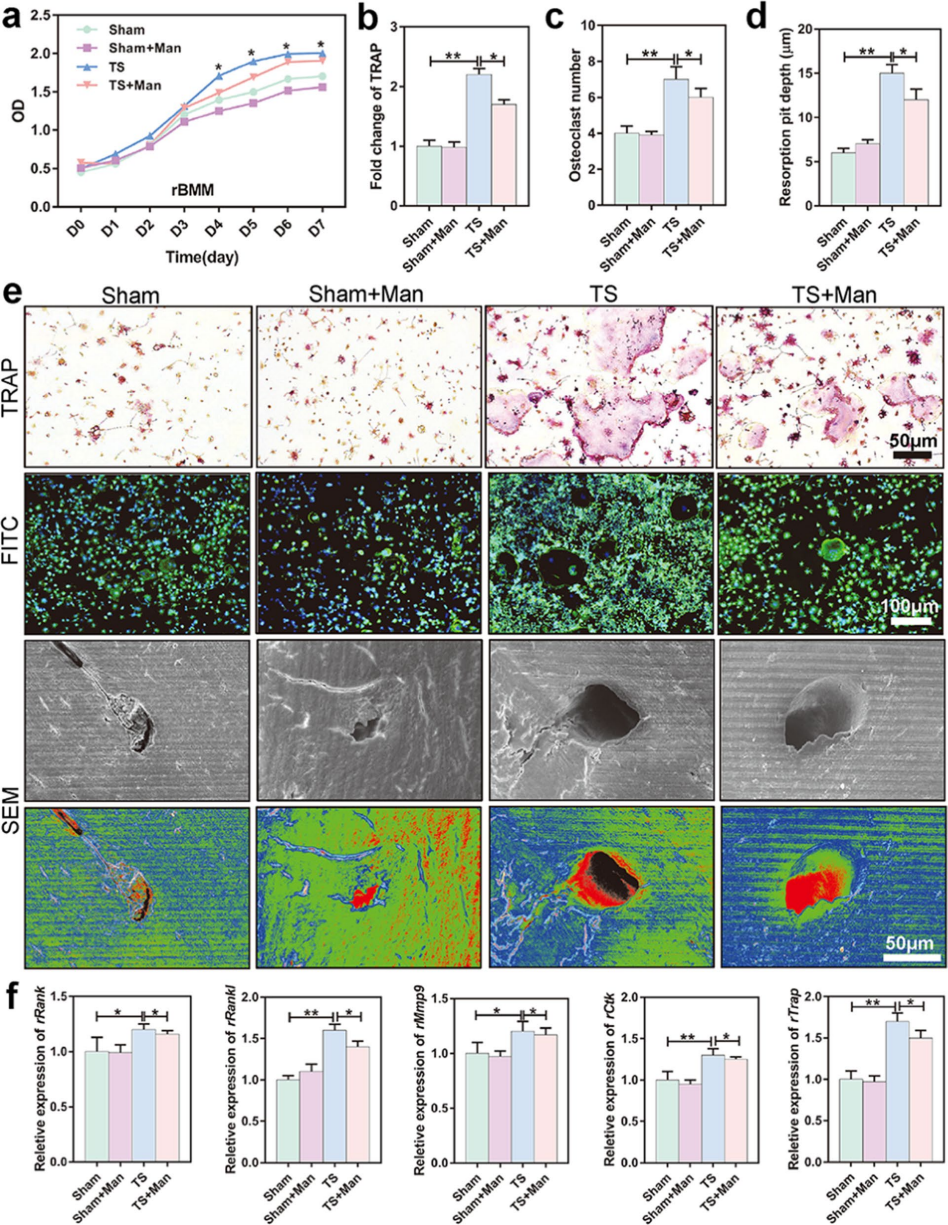
D-mannose suppressed osteoclasts proliferation and bone resorption capacity in vivo. Rat bone marrow monocytes (rBMMs) of four groups were extracted. **a** The CCK-8 proliferation curve of the rBMMs in four groups. **b** Quantification of TRAP staining results. **c** The number of osteoclasts were counted in FITC immunofluorescence staining. **d** Bone pit absorption depth in SEM. **e** Representative TRAP staining images. Pictures on the second line are representative FITC immunofluorescence staining. The blue ones are the nuclei stained by DAPI and the green ones are the cell membranes stained by FITC. The giant cells with more than three nuclei are osteoclasts. Pictures on the third and fourth line are representative SEM images of bone grinding slice. Bar represents 100 μm and 50 μm, respectively. **f** Relative expression of osteoclastogenesis-relating genes including *rRank*, *rRankl*, *rMmp9*, *rCtk*, and *rTrap*. Data are expressed as mean ± SD

The proliferation curve of rBMMs was significantly higher in the TS group than in the Sham and Sham + Man groups; notably, D-mannose inhibited the proliferation of rBMMs (TS + Man *vs*. TS group, *P* = 0.016, *P* = 0.028, *P* = 0.011, *P* = 0.027, respectively) (Fig. 5a), consistent with the in vitro results. Figure 5e shows representative images of TRAP and FITC staining (Additional file 1: Fig. S7), as well as SEM of rBMMs. The number of TRAP-stained osteoclasts was significantly increased in the TS group (*P* < 0.001), whereas D-mannose suppressed osteoclast proliferation in the TS + Man group (*P* = 0.014, *P* = 0.012, respectively) (Fig. 5b, c). Notably, resorption pit depth was deeper in the TS group and shallower in the TS + Man group (*P* = 0.015) (Fig. 5d). Furthermore, the levels of osteoclastogenesis-associated mRNAs (e.g., *Rank*, *Rankl, Mmp9, Ctk,* and *Trap*) were increased in the TS group, and decreased in the TS + Man group (Fig. 5f). These results indicated that osteoclast formation and bone resorption activity were increased in rats exposed to simulated microgravity, and D-mannose inhibited these changes in vivo. The findings in rBMMs confirmed the inhibitory effect of D-mannose on osteoclastogenesis.

### D-mannose had no apparent effect on osteogenesis in vitro

**Fig. 6:**
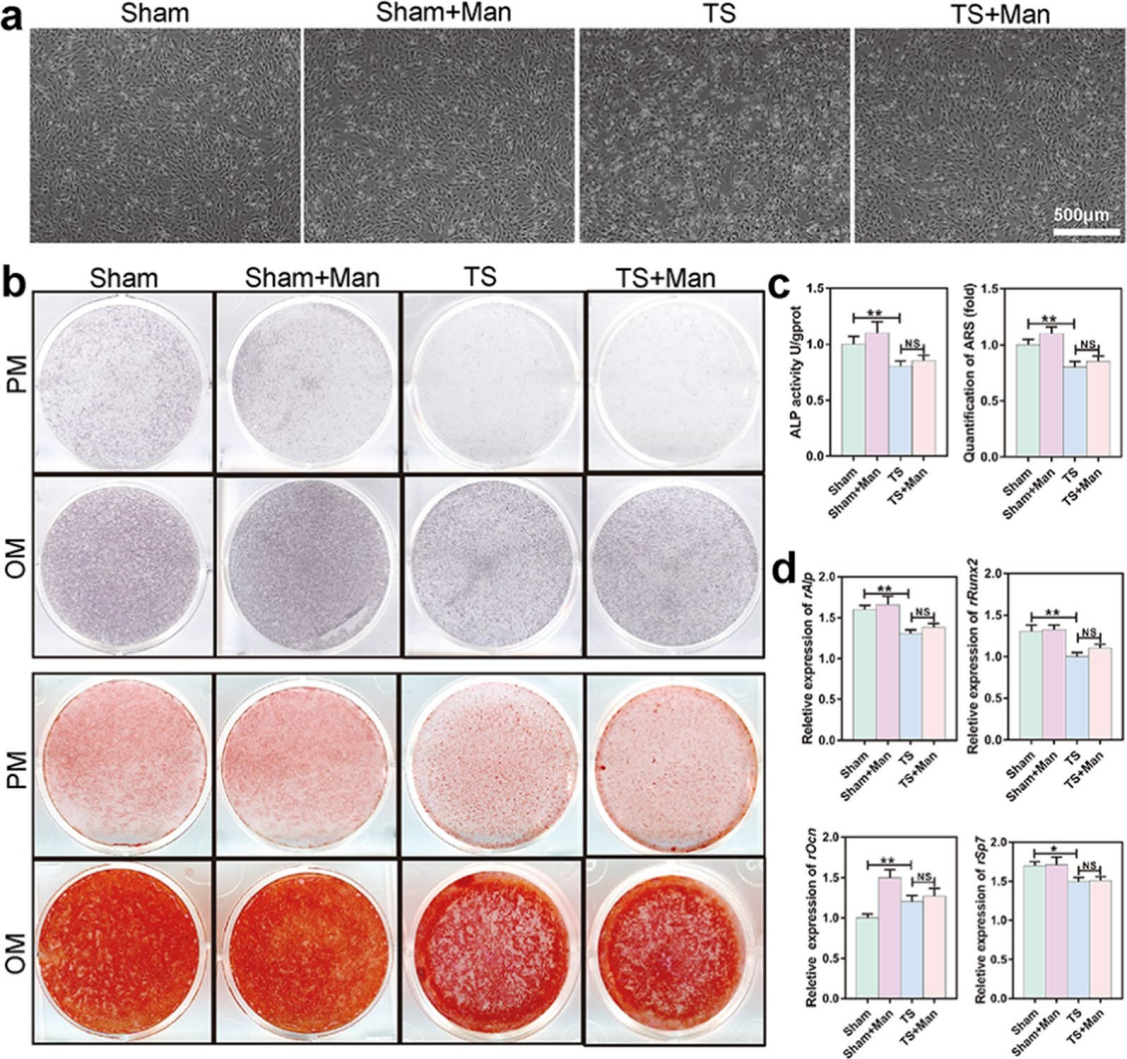
D-mannose exhibited no obvious effect on osteogenesis in vivo*.* **a** The representative optical microscope images of rat bone marrow stroma cells (rMSCs) in four groups. **b** The representative images of ALP (the above two lines) and ARS (the below two lines) stain. **c** Quantification of ALP and ARS. **d** Relative expression of osteogenesis-relating genes including *rAlp*, *rRunx2*, *rOcn*, and *rSp7*. Data are expressed as mean ± SD

Bone homeostasis is maintained by a balance of osteoclastogenesis and osteogenesis. Thus, to investigate whether D-mannose could influence osteogenesis ex vivo, we extracted rat bone marrow stromal cells (rMSCs) from each of the four groups. Representative optical microscope images of all rMSCs are shown in Fig. 6a. ALP and ARS staining revealed weaker osteogenic ability in rMSCs from the TS group compared to the Sham group; however, osteogenic ability in rMSCs did not significantly differ between the TS and TS + Man groups (Fig. 6b). Quantitative analysis of ALP staining and semi-quantitative analysis of ARS staining demonstrated that osteogenic ability was significantly weaker in rMSCs from the TS group compared to the Sham group (*P* < 0.001), but osteogenic ability in rMSCs did not significantly differ between the TS and TS + Man groups (Fig. 6c). Furthermore, the levels of osteogenesis-associated mRNAs (e.g., *Alp, Runx2, Ocn*, and *Sp7*) were lower in the TS group than in the Sham group (*P* < 0.001 for *Alp, Runx2* and *Ocn*; *P* = *0.029* for *Sp7*); however, these mRNA levels did not significantly differ between the TS and TS + Man groups (Fig. 6d).

### D-mannose inhibited osteoclastogenesis through dendritic cell-specific transmembrane protein (DC-STAMP)-mediated osteoclast fusion

**Fig. 7:**
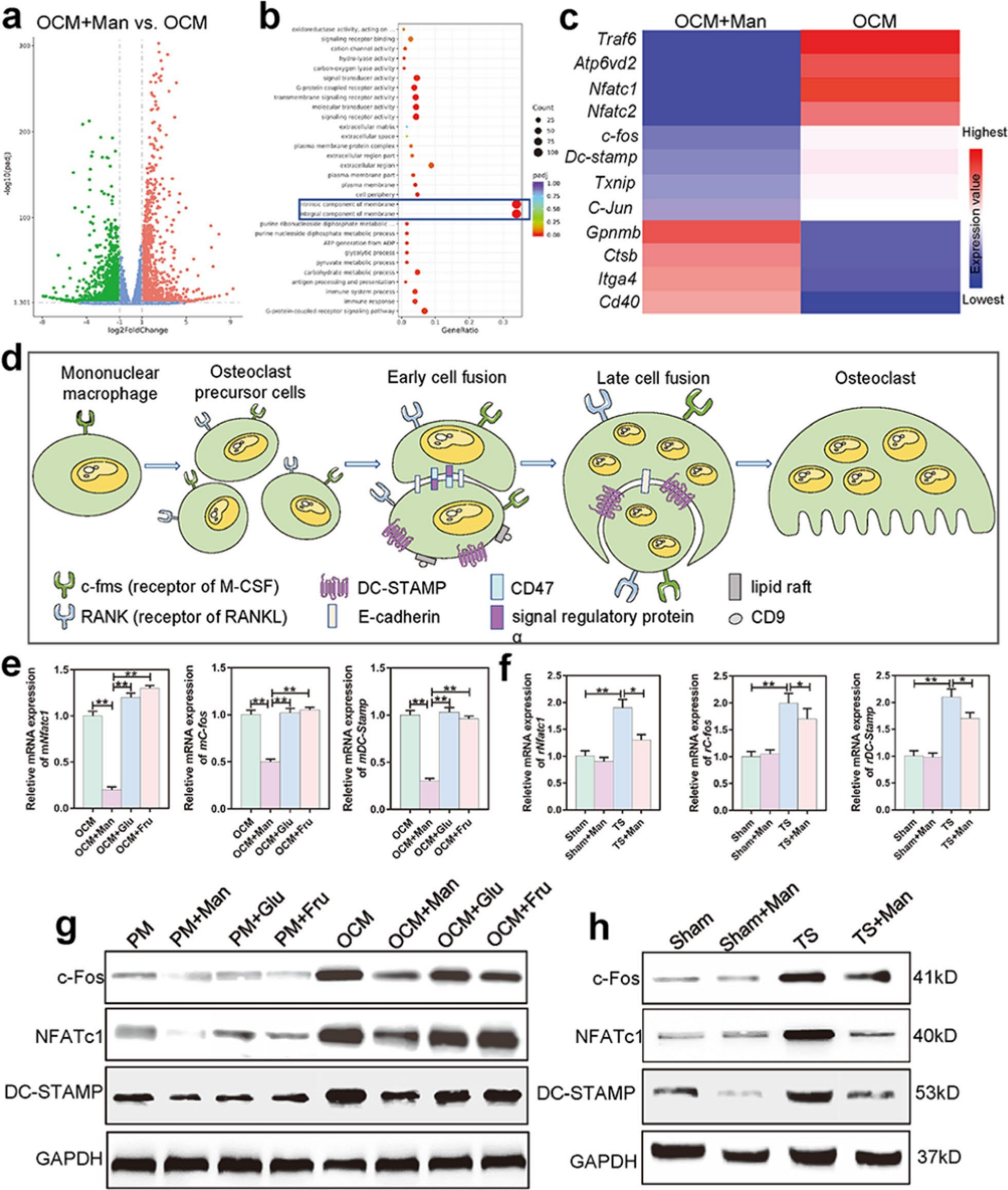
D-mannose restrained osteoclastogenesis through DC-STAMP mediated osteoclast fusion. **a** Volcanic map of RNA sequencing. The green dots on the left indicated there were 475 downregulated RNAs and 544 upregulated RNAs in OCM + Man group compared with OCM group. **b** GO gene enrichment map. **c** Gene expression heat map. **d** Schematic illustration of osteoclast fusion mechanism. **e** Relative expression of osteogenesis-relating genes including *mNfatc1*, *mC-fos,* and *mDc-stamp* of RAW264.7 cells. **f** Relative expression of osteogenesis-relating genes including *rNfatc1*, *rC-fos,* and *rDc-stamp* of rBMMs. **g** Western blot analysis of NFATc1, c-Fos, and DC-STAMP expression of RAW264.7 cells. **h** Western blot analysis of NFATc1, c-Fos, and DC-STAMP expression of rBMMs

To explore the potential mechanism by which D-mannose inhibits osteoclast formation, we performed RNA-seq transcriptomic analysis. As shown in Fig. 7a, there were 475 downregulated RNAs and 544 upregulated RNAs in the OCM + Man group, compared to the OCM group. Gene Ontology (GO) gene enrichment maps showed that these altered genes were directly and indirectly associated with cell membrane components (Fig. 7b). Gene expression heat map analysis confirmed that D-mannose downregulated the expression levels of genes associated with osteoclast fusion, such as *Nfatc1, c-Fos,* and *Dc-stamp* (Fig. 7c). Figure 7d exhibited a schematic diagram of osteoclast formation, in which cell fusion is a crucial step.

During in vitro differentiation of RAW264.7 cells into osteoclast-like cells, the expression levels of osteoclast fusion-associated mRNAs (e.g., *Nfatc1, c-Fos*, and *Dc-stamp*) were increased in the OCM group, but decreased in the OCM + Man group (*P* < 0.001). However, the presence of glucose or fructose at the same concentration did not produce an outcome similar to the effect of D-mannose (Fig. 7e). Furthermore, western blotting revealed that the expression levels of osteoclast fusion-associated proteins (e.g., NFATc1, c-Fos, and DC-STAMP) were increased in the OCM group, but decreased in the OCM + Man group. The presence of glucose or fructose at the same concentration did not produce an outcome similar to the effect of D-mannose (Fig. 7g). Notably, rBMMs extracted from rats showed similar results. The relative expression levels of *Nfatc1, c-Fos,* and *Dc-stamp* were increased in the TS group (*P* < 0.001), but decreased in the TS + Man group (*P* = 0.014, *P* = 0.013, *P* = 0.018, *P* = 0.022, respectively) (Fig. 7f). Western blotting also showed that the expression levels of NFATc1, c-Fos, and DC-STAMP were increased in the TS group and decreased in the TS + Man group (Fig. 7h).

## Discussion

It is widely acknowledged that bone homeostasis is maintained and orchestrated by osteoclast-mediated bone resorption and osteoblast-mediated bone formation [53]. Abundant studies have shown that under the influence of the space microgravity environment, bone homeostasis is unbalanced and osteoclast absorption becomes active, ultimately leading to bone loss [54–56]. Our results are consistent with these findings. Histological staining and analysis of serum indicators of osteogenesis and osteoclastogenesis in the present study indicated that both enhanced osteoclast-mediated bone resorption and hampered osteoblast-mediated bone formation occurred in TS rats. Presently, the main prevention measures of osteoporosis induced by weightlessness in astronauts are physical exercise [57] and drug intervention [58]. Physical exercise can improve blood circulation and the microstructure of bones. Common methods of physical exercise include treadmill exercise [58], bicycle calorimeter exercise, and wearing a penguin suit [59]. Other measures are also used to treat bone loss [60–62]. However, these methods are mainly aimed at promoting bone formation and have little effect on inhibiting osteoclast resorption. Commonly used drugs for the treatment of weightless induced osteoporosis include bisphosphonates, parathyroid hormone, calcitonin and traditional Chinese medicine. However, most tested compounds had side effects, such as digestive and renal disturbance, and hepatic dysfunction [15–17].

Cranberries have been reported to attenuate periodontal bone loss but whether cranberries have a protective effect against bone loss in microgravity remains unclear. In the present study, we used the TS rats, a classic animal model simulating microgravity [43], to mimic weightlessness on Earth. Not unexpectedly, significant bone loss occurred in load-bearing bones including femurs and vertebrae in TS rats, consistent with previous findings [63]. Interestingly, supplementation with cranberries and blueberries but not raspberries supplement significantly improved microgravity-induced bone loss in rats. HPLC was used to analyze the active ingredients in cranberries and blueberries. The D-mannose content was lowest in raspberries, which also demonstrated the weakest reversal of bone loss, indicating that the osteoprotective effect of cranberries was partly caused by D-mannose. Although differences in D-mannose content were found between cranberries and blueberries, the osteoprotective effect did not significantly differ between these two fruits, possibly because D-mannose acts as a beneficial health product over a wide range of concentrations; it may be ineffective only at very low concentrations, such as in raspberries. Thus, we inferred that the D-mannose in cranberries and blueberries conferred protective effects against bone loss. Additionally, although the contents of anthocyanins and proanthocyanidins were relatively high among the three fruits, there were no differences in their contents (Additional file 1: Fig. S2), which was inconsistent with the different levels of bone protection conferred by the three fruits. This further confirmed the role of D-mannose in the fruit.

Our previous studies revealed that D-mannose could attenuate bone loss both in senile and ovariectomized mouse models mimicking aging and estrogen deficiency osteoporosis, respectively [41]. Nevertheless, the effects of D-mannose on bone metabolism in space have not been explored. Therefore, we investigated whether D-mannose could have osteoprotective effects in space using TS rats. We used D-mannose concentrations of 1.1 M in vivo and 25 mM in vitro, referring to the research of Zhang et al.[39]. Additionally, the anti-inflammatory effects of both low and high concentrations of D-mannose were explored in our preceding studies, which showed that D-mannose dramatically upregulated the percentage of Treg cells independently of the concentrations [41].The present study suggests that D-mannose could attenuate weightlessness-induced bone loss in weight-bearing bones such as femurs and vertebrae. Notably, the mandibular BMD was similar in the TS and Sham groups (Additional file 1: Fig. S3), presumably because the local masticatory force of the jaw prevented mandibular bone loss regardless of body position. Thus, only load-bearing bones (e.g., femurs and vertebrae) exhibited disuse-induced bone loss.

Bone metabolism includes both osteogenesis (mediated by osteoblasts) and osteoclastogenesis (mediated by osteoclasts) [64, 65]. Therefore, to understand the beneficial effects of D-mannose on bone metabolism in TS rats, we examined both osteogenesis and osteoclastogenesis in vitro and in vivo. Referring to previous studies on D-mannose [39, 41, 66], we chose D-mannose as 25 mM for our follow-up experiment and used the same concentration of glucose and fructose as the control. Notably, D-mannose suppressed osteoclastogenesis (TRAP staining of femurs; serum levels of CTX-1, a marker of osteoclast absorption) but did not promote osteogenesis (immunohistochemical staining of OCN of femurs; serum levels of P1NP, an osteogenic marker) in vivo*.* However, the presence of glucose or fructose at the same concentration did not inhibit osteoclast formation or absorption, indicating that D-mannose suppressed osteoclast proliferation and bone resorption capacity in vitro through a mechanism independent of energy metabolism.

D-mannose inhibited osteoclastogenesis, but did not promote osteogenesis. Due to difficulties associated with the isolation and ex vivo culture of osteoclasts, stimulus-induced differentiation of osteoclasts from other cell types has been increasingly used for analyses of osteoclast activity in bone diseases [67, 68]. Here, we cultured RAW264.7 cells and induced their differentiation into osteoclast-like cells through in vitro exposure to receptor activator for nuclear factor-κB ligand (RANKL) and macrophage-stimulating factor; the osteoclast-like cells were used to investigate the mechanism by which D-mannose inhibits osteoclast formation.

To further explore the inhibitory effect of D-mannose on osteoclastogenesis, RAW264.7 cells, a class of classic cells widely used in osteoclast research [68], were used. rBMMs were also cultured in vitro without RANKL for 4 days to represent the in vivo cellular state. The experimental results of both RAW264.7 cells and rBMMs revealed that D-mannose significantly inhibited proliferation, cell fusion and absorptive bone capacity of osteoclasts, which further confirmed the results of in vitro experiments. Moreover, in previous studies, we showed that D-mannose does not have an osteogenic effect on cultured human bone marrow mesenchymal stem cells in vitro [41]. Additionally, Guo et al. reported that D-mannose did not affect the osteogenic differentiation potentials of human periodontal ligament stem cells [66].

Mechanistically, RNA-seq transcriptomic analysis revealed that D-mannose administration significantly inhibited dendritic cell-specific transmembrane protein (DC-STAMP), as well as two essential transcription factors for osteoclast fusion (nuclear factor of activated T cells 1 [NFATc1] and c-Fos). The cell–cell fusion of TRAP-positive mononuclear osteoclast precursors is essential for the formation of functionally mature osteoclasts that secrete protons to resorb bone [69]. DC-STAMP is a seven-transmembrane protein specifically required for osteoclast cell–cell fusion [70]; mice deficient in DC-STAMP develop osteopetrosis due to defective osteoclast fusion [71]. DC-STAMP expression is positively regulated by NFATc1 and c-Fos, both of which are transcriptional factors essential for osteoclast differentiation [72]. In the present study, treatment of RAW264.7 cells with D-mannose inhibited expression of these fusion-related genes and proteins. The findings in extracted rBMMs were consistent with the in vitro results. Thus, D-mannose protected against bone loss in TS rats by inhibiting osteoclastogenesis via DC-STAMP-mediated osteoclast cell fusion.

UTIs are fairly common among adult women; half of all women will have ≥ 1 UTI during their lifetime and 20–40% will experience recurrent UTIs [35]. However, studies of UTIs in space or microgravity are limited. In the present study, H&E staining revealed a large zone of infiltrating inflammatory cells in renal tissue from rats in the TS group. D-mannose is widely regarded as a treatment for UTIs [35, 73, 74]. Here, we found that UTI symptoms were reduced in the TS + Man group based on histological staining of renal and urethral tissue, as well as inflammatory cell counts. Due to high recurrence ratios and the incremental degree of antibiotic resistance among uropathogens, UTIs constitute a grievous health problem. D-mannose, a monosaccharide, can suppress bacterial adhesion to the urothelium.

## Limitations

Several limitations of our study should be further addressed. Whether different concentrations of D-mannose exhibited different effects on osteoclasts should be further explored, although D-mannose may act as a beneficial health product over a wide range of concentrations. The concrete mechanisms of D-mannose in osteoclast cell fusion should also be thoroughly investigated if D-mannose is to be used in astronauts to combat weightlessness-induced bone loss and UTIs during space missions in the future.

## Conclusion and outlook

The present findings demonstrate that TS rats in a simulated microgravity environment experienced severe bone loss because of disrupted bone homeostasis as well as UTIs because of dysuria. Cranberries could protect against bone loss and UTIs in TS rats, and the D-mannose present in cranberries had important osteoprotective and anti-inflammatory effects. Both in vivo and in vitro experiments showed that D-mannose blocked osteoclastogenesis via DC-STAMP-mediated osteoclast cell fusion. Our results may be useful in designing a safe strategy for protection against bone loss and UTIs in astronauts during space missions.

## Supplementary Information

**Additional file 1**: **Figure S1**. a) A rat tail suspension device. b) The rats were suspended from the tail. c) Juice from three kinds of fruit: cranberry, blueberry, and raspberry. d) Implementation of animal experiments. e) Curve graph of body weight of rats over time. **Figure S2**. Anthocyanins in three fruits were determined by HPLC. HPLC: high performance liquid chromatography. **Figure S3**. Suspension does not cause bone loss in rat jaw. a) Representative micro-CT images of mandibular bone. b) Bone mineral density was analyzed at the root bifurcation of the first mandibular molar (red arrow) and mandibular angle (red circle). c) Bone mineral density of alveolar and mandibular angle. **Figure S4**. D-mannose alleviated urinary tract infections in rats exposed to simulated microgravity. a) H&E staining of kidney and urethra slice and blue arrows indicate hemorrhagic foci. b) Count of leukocytes in urine and neutrophils in blood of rats. **Figure S5**. D-mannose supplement exhibited no obvious toxic and side effects on the body of rats. H&E staining of slice of liver, spleen, and kidney. **Figure S6**. Fluorescein isothiocyanate staining of RAW 264.7 cells. **Figure S7**. Fluorescein isothiocyanate staining of rat bone marrow-derived macrophages (rBMDMs).

## Data Availability

All data generated and analyzed during the current study are contained within the manuscript and are available from the corresponding author on reasonable request.

## Abbreviations

TS: Tail suspend
HPLC: High performance liquid chromatography
Tb.BMD: Bone mineral density in trabecular bone
BV/TV: Bone volume/total volume ratio
Tb.Th: Trabecular thickness
Tb.N: Trabecular number
BS/BV: Bone surface area/bone volume ratio
Tb.Sp: Trabecular separation
H&E: Hematoxylin and eosin
TRAP: Tartrate-resistant acid phosphatase
OCN: Osteocalcin
CTX-1: C-terminal telopeptide of collagen type 1
P1NP: Procollagen type I N-terminal propeptide
PM: Preliminary culture medium
OCM: Osteoclast medium
OCM + Man: Osteoclast medium + 25 mM D-mannose
OCM + Glu: Osteoclast medium + 25 mM glucose
OCM + Fru: Osteoclast medium + 25 mM fructose
DAPI: 4',6-Diamidino-2-phenylindole
FITC: Fluorescein isothiocyanate isomer
SEM: Scanning electron microscope
RANK: Receptor activator of nuclear kappa-B
RANKL: Receptor activator of nuclear kappa-B ligand
Mmp9: Matrix metalloproteinase 9
Ctk: Cathepsin K
ALP: Alkaline phosphatase
ARS: Alizarin Red S
Runx2: Runt-related transcription factor 2
Ocn: Osteocalcin
Sp7: Specificity protein 7
DC-STAMP: Dendritic cell-specific transmembrane protein
NFATc1: Nuclear factor of activated T-cells
