# Supplementary material for: D-Mannose prevents bone loss under weightlessness
Source: J Transl Med. 2023 Jan 9;21:8. doi: 10.1186/s12967-022-03870-1 (PMC9827691; doi:10.1186/s12967-022-03870-1)
Supplement: Supplementary file 1 — Additional file 1: Figure S1. a) A rat tail suspension device. b) The rats were suspended from the tail. c) Juice from three kinds of fruit: cranberry, blueberry, and raspberry. d) Implementation of animal experiments. e) Curve graph of body weight of rats over time. Figure S2. Anthocyanins in three fruits were determined by HPLC. HPLC: high performance liquid chromatography. Figure S3. Suspension does not cause bone loss in rat jaw. a) Representative micro-CT images of mandibular bone. b) Bone mineral density was analyzed at the root bifurcation of the first mandibular molar (red arrow) and mandibular angle (red circle). c) Bone mineral density of alveolar and mandibular angle. Figure S4. D-mannose alleviated urinary tract infections in rats exposed to simulated microgravity. a) H&E staining of kidney and urethra slice and blue arrows indicate hemorrhagic foci. b) Count of leukocytes in urine and neutrophils in blood of rats. Figure S5. D-mannose supplement exhibited no obvious toxic and side effects on the body of rats. H&E staining of slice of liver, spleen, and kidney. Figure S6. Fluorescein isothiocyanate staining of RAW 264.7 cells. Figure S7. Fluorescein isothiocyanate staining of rat bone marrow-derived macrophages (rBMDMs). [file 12967_2022_3870_MOESM1_ESM.docx]

**D-Mannose Prevent Bone Loss under Weightlessness**


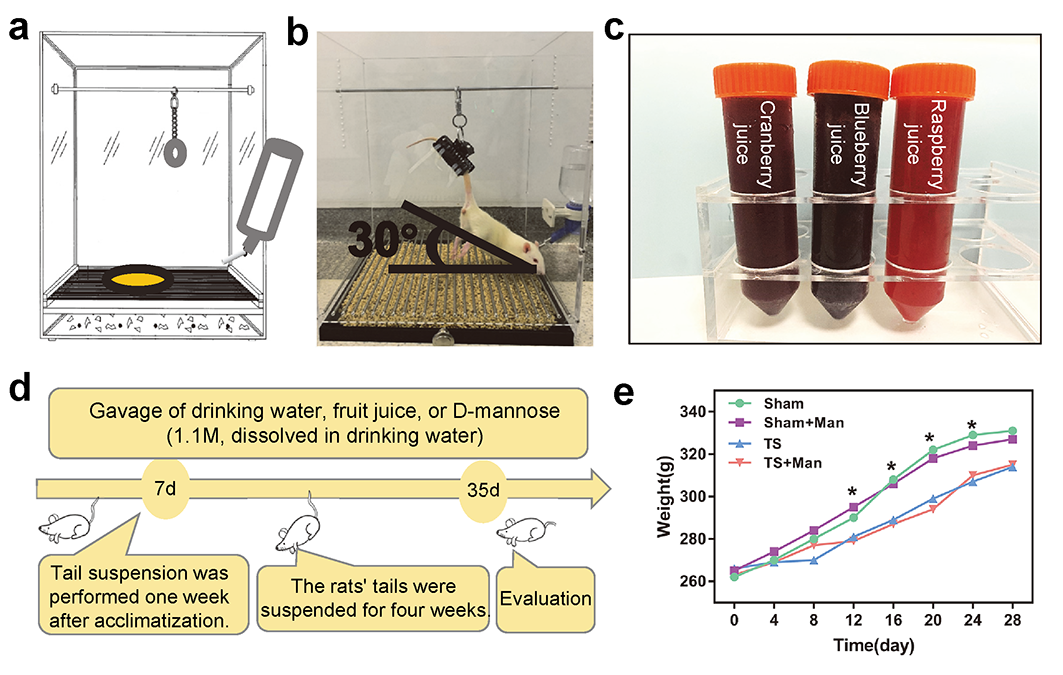


**Additional file 1: Fig. S1.** a) A rat tail suspension device. b) The rats were suspended from the tail. c) Juice from three kinds of fruit: cranberry, blueberry, and raspberry. d) Implementation of animal experiments. e) Curve graph of body weight of rats over time.


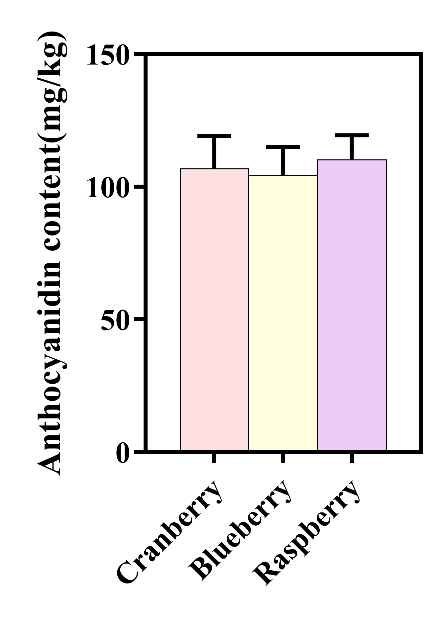


**Additional file 1: Fig. S2.** Anthocyanins in three fruits were determined by HPLC. HPLC: high performance liquid chromatography.


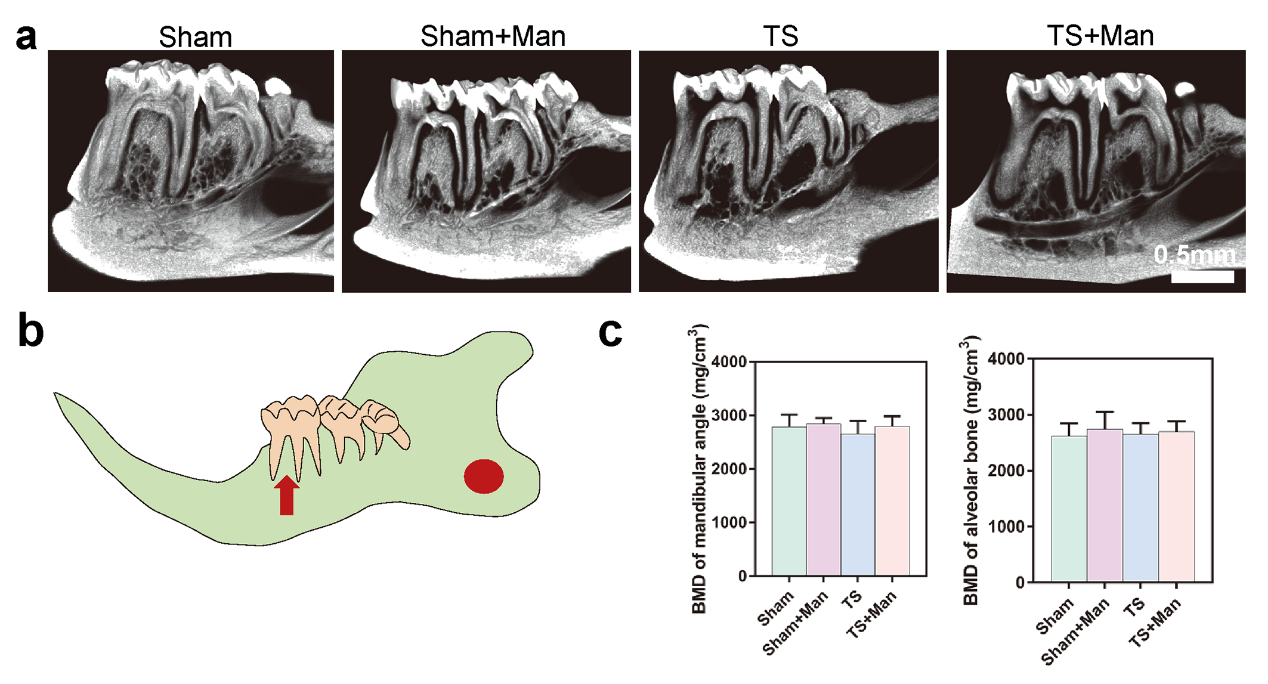


**Additional file 1: Fig. S3.** Suspension does not cause bone loss in rat jaw. a) Representative micro-CT images of mandibular bone. b) Bone mineral density was analyzed at the root bifurcation of the first mandibular molar (red arrow) and mandibular angle (red circle). c) Bone mineral density of alveolar and mandibular angle.


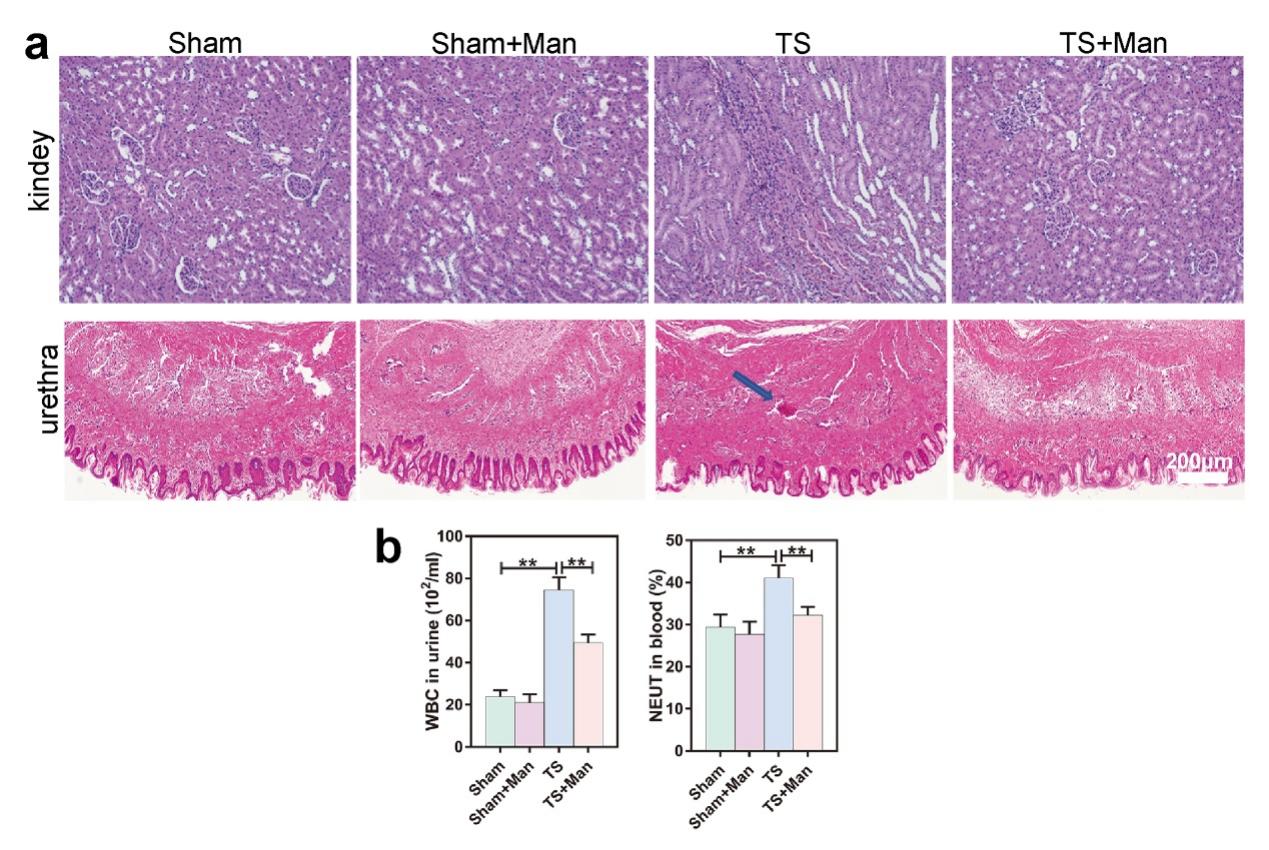


**Additional file 1: Fig. S4.** D-mannose alleviated urinary tract infections in rats exposed to simulated microgravity. a) H&E staining of kidney and urethra slice and blue arrows indicate hemorrhagic foci. b) Count of leukocytes in urine and neutrophils in blood of rats.


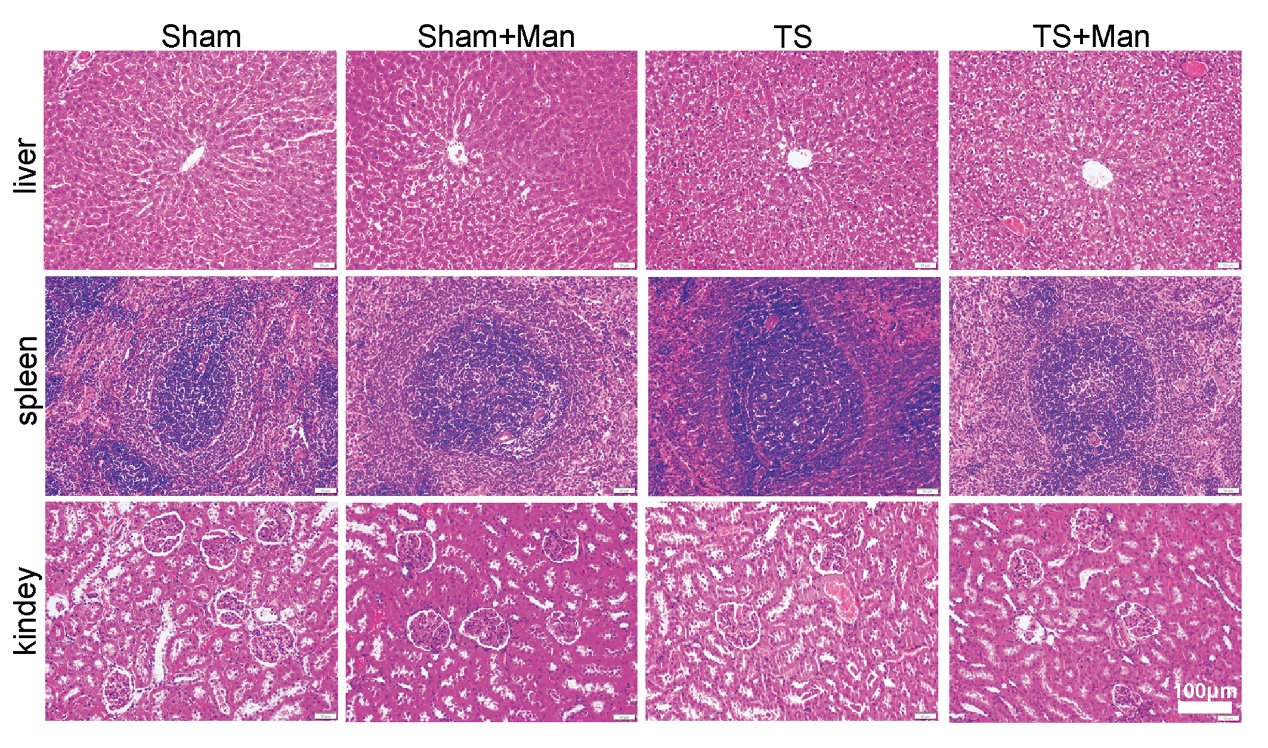


**Additional file 1: Fig. S5.** D-mannose supplement exhibited no obvious toxic and side effects on the body of rats. H&E staining of slice of liver, spleen, and kidney.


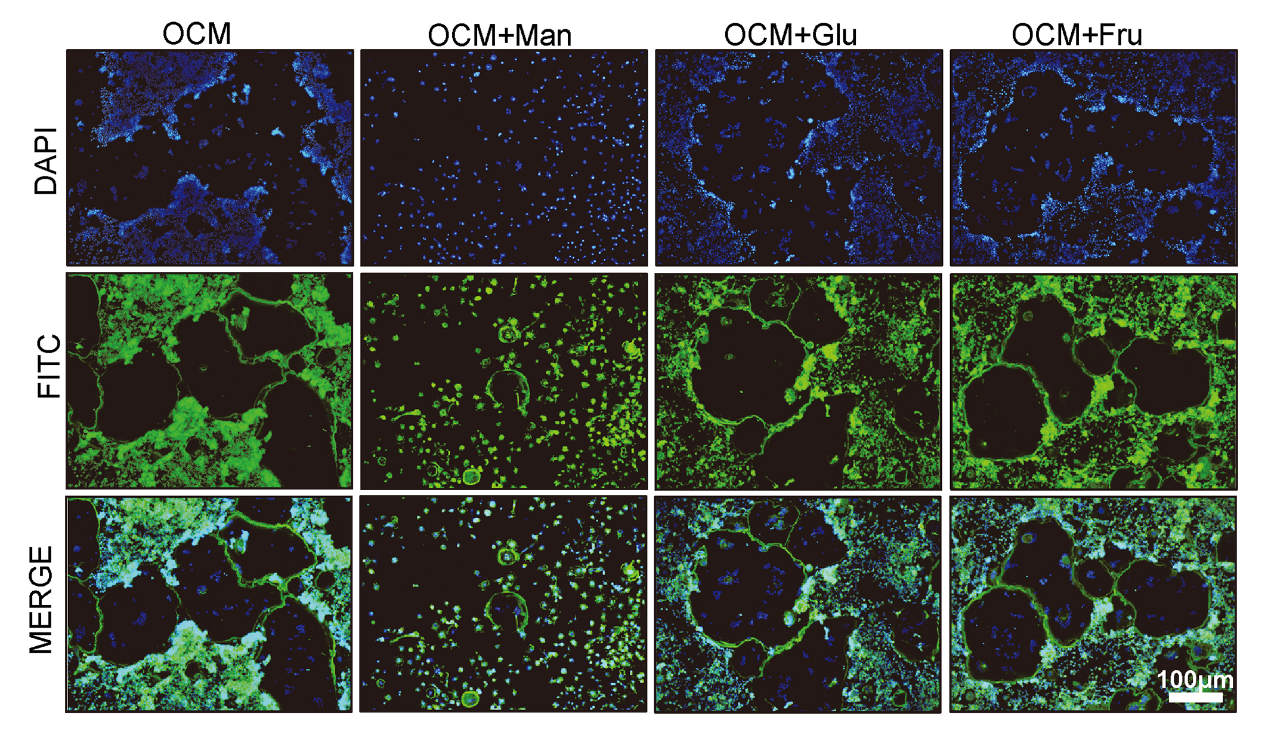


**Additional file 1: Fig. S6.** Fluorescein isothiocyanate staining of RAW 264.7 cells.


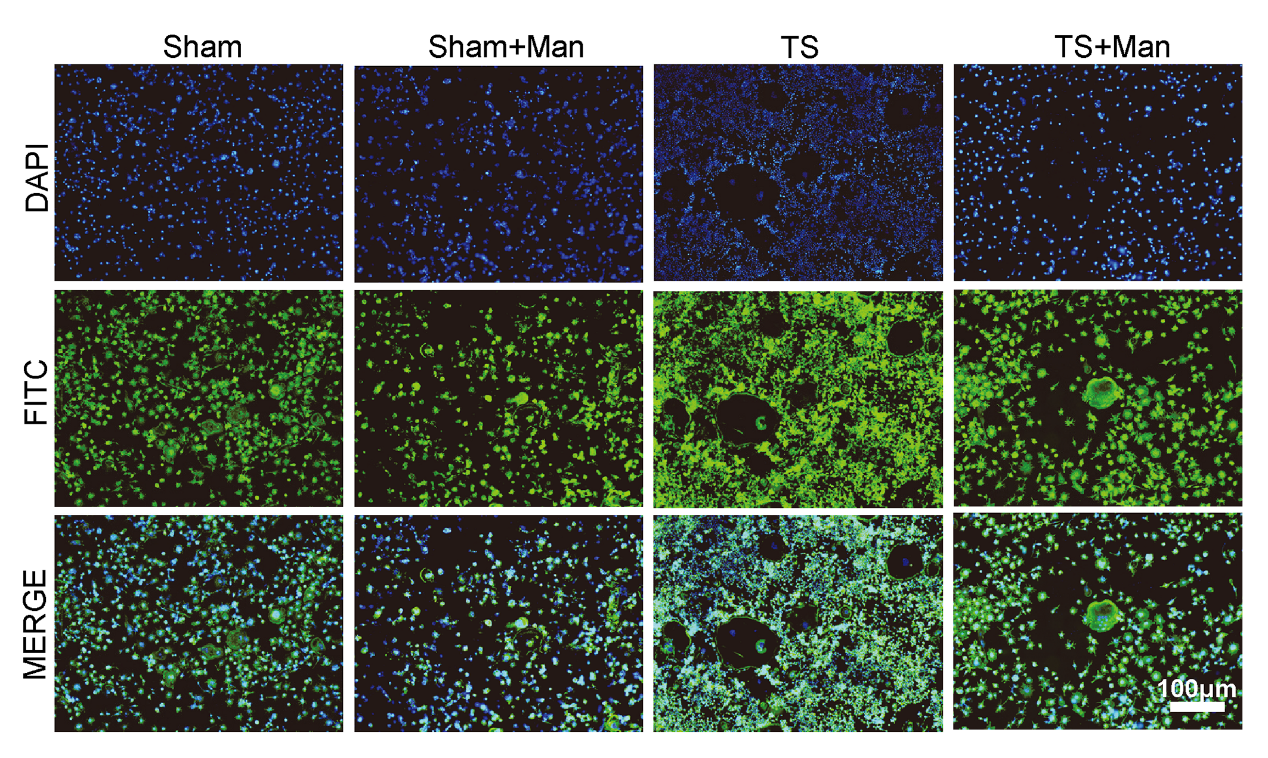


**Additional file 1: Fig. S7.** Fluorescein isothiocyanate staining of rat bone marrow-derived macrophages (rBMDMs).
